# The Association between Index of Nutritional Quality (INQ) and Obesity: Baseline Data of Kharameh Cohort

**DOI:** 10.1155/2022/8321596

**Published:** 2022-11-17

**Authors:** Maryam Jalali, Parisa Keshani, Masoumeh Ghoddusi Johari, Ramin Rezaeianzadeh, Seyed Vahid Hosseini, Abbas Rezaianzadeh

**Affiliations:** ^1^Colorectal Research Center, Shiraz University of Medical Sciences, Shiraz, Iran; ^2^Breast Diseases Research Center, Shiraz University of Medical Sciences, Shiraz, Iran; ^3^Experimental Medicine Program, Department of Medicine, Faculty of Medicine, University of British Columbia, Vancouver, British Columbia, Canada

## Abstract

Obesity is an increasing problem that can lead to noncommunicable diseases. The role of dietary factors on one's obesity is confirmed in many studies. One nutritional approach that can be used for assessment of the foods and diets is the Index of Nutritional Quality (INQ). Our study is aimed at exploring the association between INQ and obesity. Our hypothesis is that enriched and high-quality diets reduce the risk of overweight or obesity. This study was carried out on 6248 overweight and obese participants, from whom 4356 (69.7%) and 1892 (30.3%) were overweight and obese, respectively. To assess the dietary intake for the participants, a valid food frequency questionnaire (FFQ) with 130 food items was utilized. The analysis revealed an inverse association between the overweight and the INQ of iron, thiamin, riboflavin, B6, folate, zinc, magnesium, calcium, and vitamin C and E. For the obese group, this inverse association was found for iron, B6, folate, zinc, magnesium, calcium, and vitamin C and E. These results approved our hypothesis that a rich nutrition diet may lead to a lower risk of obesity.

## 1. Introduction

Obesity is an increasing problem that has recently affected both developed and developing countries and can lead to noncommunicable diseases including diabetes, heart disease, and lower life expectancy [1–4]. According to the World Health Organization (WHO), there are more than one billion overweight people in the world, 300 million of whom are reported as obese people [5–7].

Based on recent studies, a nutrition transition has happened in Iran [8]. In this period, the dietary pattern and physical activity of people have changed; their diet is rich in sugar and fats, and fiber consumption has reduced. Therefore, overweight and obesity have increased [8, 9]. In Iran, the prevalence of overweight is reported about 22% and 40% among the 15–39 and 40–69 age category, respectively [8].

The role of dietary factors on the individuals' obesity was discussed in previous studies [10–13]. While a variety of methods exist for dietary intake analysis, it is proposed that the tools for accessing the overall dietary quality should be applied rather than the nutrients separately. The underlying deduction is that analyzing dietary consumption as a total index is more straightforward than inspecting every dietary component [14].

One nutritional approach that can be used for qualitative and quantitative assessment of the foods and diets is the Index of Nutritional Quality (INQ). One prominent advantage of INQ is that by applying this method, single diets, meals, and food can be analyzed both qualitatively and quantitatively [14–16]. In addition, in comparison to the alternative methods, the effect of energy intake is adjusted in INQ computation [17]. INQ is a fraction whose numerator and denominator are subsequently each nutrient intake percentage and the average requirement percentage of the diet calorie [15]. The INQ is simple and accurate in comparison to other methods because it adjusts the effects of energy intake.

Considering the mentioned consequences of obesity on the quality of life [1–4, 18, 19] and consensus of the recent researches about the preventability of most types of obesity [20], we aimed to examine the association of the INQ, as a comprehensive index to assess the intake quality of micronutrients, and obesity in overweight and obese participants with an age range of 40-70 years old in a cross-sectional and population-based study.

## 2. Material and Methods

### 2.1. Participants

Overall, after applying the inclusion and exclusion criteria of our study, 10439 subjects with an age range of 40-70 years old participated in this population-based cross-sectional study in Kharameh between 2014 and 2017. Kharameh is one of the southern cities of Fars Province with 61,580 people. Kharameh cohort study is a part of the Prospective Epidemiological Research Studies in Iran (PERSIAN cohort study). The main aim of the PERSIAN study is to find the incidence and risk factors of noncommunicable diseases, and its rationale and design have been published in detail (22). Kharameh study was started with 10,667 participants with an age range of 40–70 in 2014. All participants of Kharameh cohort study were included in our research through census [21].

Individuals with mental retardation and inability and reluctance to take part in the study were excluded from the study. The participants whose total daily energy intake (Kcal) was in the range of mean ± 3SD were considered eligible, and the others were excluded [22, 23]. Finally, data analysis was done on 6248 participants, and written informed consent was provided for all the participants.

### 2.2. Measurement

Demographic information was gathered through a standardized questionnaire, the validity and reliability of which was checked by the national team of PERSIAN cohort. The participants' weight was measured using SECA Germany scale with light clothing and without shoes. Body mass index (BMI) was categorized as less than 25 (normal weight), between 25 and 29 (over weight), and equal to and more than 29 (obese) [24].

### 2.3. Dietary Assessment

To assess the dietary intake of the participants, a valid food frequency questionnaire (FFQ) with 130 food items was utilized [25]. A face-to-face interview was performed, and the amount of the consumption of each food item was asked in a period of one past year as daily, weekly, and monthly. Finally, all the data were collected in the form of daily intake, and Nutritionist IV software, which had been modified for Persian foods (version 7.0; N-Squared Computing, Salem, OR, USA) based on Iranian food composition tables, was applied to analyze the individuals' dietary intake for their energy content and micronutrients [25] in order to calculate the INQs.

### 2.4. Physical Activity Assessment

In order to assess the physical activity including the sleeping duration, sport, and occupation in a day, a validated physical activity questionnaire was applied; based on the questionnaire instruction, the Metabolic Equivalent Task (MET) index was computed based on units per hour per day [25].

### 2.5. Assessment of INQ

The INQ is a technique for analyzing the diets and foods that have particular importance in assessment of issues related to clinical nutrition. The INQ is computed as the “ratio of nutrient-to-calorie content of foods”. In calculation of INQ, the standard amount and the number of nutrients are the flexible parameters that can vary in each clinical case [16, 26–28] definition. To calculate the INQ scores for each participant, the dietary data (energy and micronutrients) were derived from the FFQ using Nutritionist IV software. Then, the data were calculated in 1000 kcal of energy for each micronutrient. Finally, the INQs were calculated based on the following definition: INQ is the division of nutrient amount in 1000 kcal to its Recommended Dietary Allowance (RDA) in 1000 kcal [29]. The micronutrients used in calculating the INQs were vitamin A, vitamin C, vitamin D, vitamin E, vitamin B6, vitamin B12, iron, folate, copper, magnesium, zinc, calcium, selenium, thiamin, riboflavin, niacin, and pantothenic acid.

### 2.6. Statistical Analyses

A chi-squared or Fisher's exact test and independent sample *t*-test were applied for between group comparison of the qualitative and quantitative variables subsequently. For the quantitative variable, Mann–Whitney test was used in the case of nonnormality. Binary logistic regression was applied for calculation of the odds ratio and its 95% confidence intervals. All the analysis was done in IBM SPSS software (version 21), and a *P* value <0.05 was considered as the significant level. In this study, obese and overweight participants were considered as the groups, and sex, age, physical activity, marital status, having a job, and years of education were considered as the covariates in investigating the association between INQ and obesity.

## 3. Results

Altogether, there were 6248 overweight and obese participants, from whom 4356 (69.7%) and 1892 (30.3%) were overweight and obese, respectively.  Table 1 shows the demographic information of the participants and the INQ. Additionally, the comparison of the INQ between obese and overweight subjects is displayed in this Table.

Table 2 shows the ORs and 95% confidence intervals of logistic regression after adjusting for the covariates as age, gender, education year, marital status, and physical activity.

According to the results of logistic regression that modeled the relation between risk of obesity (overweight) as a binary dependent variable and INQs of the nutrition as independent continuous variables after adjusting for the covariates as age, gender, education year, marital status, physical activity, and having job, inverse association between being overweight and INQ of iron, thiamin, riboflavin, B6, folate, zinc, magnesium, calcium, and vitamins C and E was identified.(all *P* value <0.05).

Similarly, an inverse association was found between being obese and the INQ of iron, B6, folate, zinc, magnesium, calcium, and vitamins C and E after adjusting for covariates. Briefly, it means that a lower level for the INQs increases the risk of overweight and obesity.

## 4. Discussion

In our population-based study, the associations between the INQS and obesity and overweight were investigated. The analysis revealed the inverse association between being overweight and the INQ of iron, thiamin, riboflavin, B6, folate, zinc, magnesium, calcium, and vitamins C and E. For the obese group, this inverse association was found for iron, B6, folate, zinc, magnesium, calcium, and vitamin C and E. These results proved our hypothesis, indicating that a rich in nutrition diet can lead to lower risk of obesity.

### 4.1. Obesity and Iron Deficiency

Based on the result of our study, iron was a protective factor against obesity. The effect of iron on obesity was firstly studied in 1962 [30, 31]. Despite a high prevalence of obesity in people with iron deficiency in different age ranges, there are few studies that investigate the cause-effect association between them. In a recent study, one explanation for this relationship is the increment of hepcidin levels mediated by chronic inflammation. Also, it is stated that obesity may be a factor that disrupts iron homeostasis which leads to iron deficiency anemia [32].

The association between overweight and iron deficiency is investigated in a study by Karen et al. They stated that children and adults who were overweight or were at risk of being overweight were twice as probable to be iron deficient, compared with the normal weight. In addition, nearly one of every ten overweight adolescents suffered from iron deficiency [33].

Similarly, in a cross-sectional study with the aim of finding the association between weight and iron deficiency among children and adolescents, an association was found between being overweight and iron deficiency [34].

In another study, different factors had been diagnosed for explaining the association between overweight/obesity and iron deficiency including inadequate physical activity, genetic factors, poor and unhealthy diets, and less releasing of iron as a consequence of decreased myoglobin breakdown [33]. This mechanism for iron is indicated in a study on animals, showing that lack of iron might cause an increment in visceral adiposity [35, 36]. In addition, as the iron deficiency can cause inactivity, this may also contribute to obesity [35].

Obviously, iron deficiency is common in childbearing age, and its relation to obesity was investigated in previous studies [37]. Despite some contradictory results, [38] it is stated that reproductive age obese women had lower iron absorption compared with overweight and normal weight, that is likely because of subclinical inflammation of obesity [37].

### 4.2. Obesity and Vitamin B

Many studies confirmed the association between obesity and B vitamins [39–42]. Bernert et al. observed the lack of B vitamins in obese people [43]. In addition, studying morbidly obese women showed that 11% of them had an abnormal level of vitamin B12 [44]. However, there are some inconsistent results about the association of vitamin B12 and obesity [42, 45–48].

In another cross sectional study, the association of overweight/obesity and lack of vitamin b12 was detected. This association was the same whether the individual was male or female. The association between vitamin B12 deficiency and helicobacter pylori infection was reported in some studies [49–51], and it is also stated that helicobacter pylori infection and dyspeptic symptoms were observed commonly in obese and overweight people [52–54].

High prevalence of thiamin deficiency in certain obese populations was explored in 2015 [55]. Similarly, Via found thiamin deficiency in 15-29% of obese people [56]. In another study, higher BMI was a clinical risk factor for thiamin deficiency [57]. Despite the result of the mentioned studies, the issue of the clinical role of thiamin consumption in controlling of the obese and overweight people's weight is unclear [57]; it is hypothesized that high amounts of simple sugar in diet may lead to thiamin deficiency [57]. One common problem of obese people is carbohydrate exchange disorders. It is stated that group B vitamins may have an important role in keeping carbohydrate metabolism in normal levels, and thiamin has an important effect on this mechanism [58, 59].

Similarly, Jun et al. in 2022 found a higher rate of insufficient intake of vitamin B6 in the obese people compared to the healthy group [60].

Based on a systematic review in 2019, the serum folate in overweight and obese individuals was less concentrated in comparison with the individuals with normal weight. The explanation was that overweight and obese people have less healthy diets and consume less vegetables and fruits. In addition, obesity may be effective on folate absorption through intestinal epithelium [61].

The inverse association between some B vitamins and obesity in our study is in line with the previous studies and can be generalized to other populations.

### 4.3. Vitamin C

Vitamin C is a crucial micronutrient that should be taken by consumption of vegetables and fruits, and it is an essential item for some human body coenzyme function [62], losing weight process, and lipid metabolism [63]. Also, the role of this vitamin on the adipogenesis of preadipocytes is reported [64].

There are many studies about the inverse association of the INQ of vitamin C and obesity. For example, in a study on healthy adult girls, the INQ of vitamin C in normal weight subjects was higher than the other groups [64]. In another study, it is stated that a lower level of vitamin C was associated with higher body mass and waist circumference [65]. In a recent study with the aim of investigating vitamins C and the association with obesity in early adulthood, the intake of these vitamins after adjustment for confounders was an independent predictor of overweight/obesity. Our finding is in line with that of the mentioned studies.

Many mechanisms are suggested for the role of vitamin C in obesity. Dietary intake of this vitamin has a significant effect on the concentration of its serum [4]. The probable involvement of vitamin C in modulating adipocyte lipolysis, regulating the glucocorticoid release from adrenal glands, and inhibiting glucose metabolism and leptin secretion in adipocytes is found in previous studies. It is also expressed that this vitamin can cause reduction of glycosylation and inflammatory response and hyperglycemia improvement in diabetic obese patients [66]. In another study on Swedish children, the leptin level and vitamin C consumption are associated. Based on this study, low vitamin C concentration may alter leptin gene expression and resistance of leptin. One consequence of this outcome is the risk of obesity increment [67].

### 4.4. Obesity and Zinc, Magnesium, and Selenium

Selenium has an important biological function on our health [68]. Controlling selenium exposure in well-nourished adult populations is necessary in reducing its probable role in obesity [69]. The inverse association between dietary intake of selenium with obesity [70, 71] and overweight [72] was studied in many studies; in contrast, Correa-Rodríguez et al. found a positive association between the amount of selenium intake and risk of overweight and obesity [73]. In a recent systematic review and meta-analysis with the aim of investigating selenium status in overweight and obese participants, the frequently used markers that were applied to investigate the nutritional status of selenium in overweight and obese people were dietary intake, activity of glutathione peroxidase enzyme, and plasma/serum concentrations [74].

Magnesium is considered as a cofactor for many enzymes, so the lack of magnesium affects the metabolic syndrome and obesity. A diet rich with magnesium can be inversely associated with obesity and metabolic syndrome [75], but it is not generally true because the diet rich in it also contains other worthy elements like antioxidant vitamins and fiber [68]. There are many cross-sectional and case-control studies that have found a negative relationship between obesity/overweight and magnesium [70, 72, 76, 77].

Zinc is another essential element that contributes to all enzyme activities [68]. There are many studies about the negative effect of zinc on body fat mass [78–81]; however, some of them did not find any association between them [77, 82]. Our findings about the inverse effect of these three elements of dietary intake on overweight and obese people are in the same line with the mentioned studies.

Although an inverse association between the INQs and overweight/obesity was found in our study, the accurate mechanism of the effect of some of them like zinc, iron, selenium C, vitamin B5, and vitamin B6 on obesity is still unclear. The mechanism for some of them can be justified to some extent; for example, the reason for the inverse association of zinc and obesity is probably the fact that zinc is used in the most of the metabolic functions of human body. It is found that zinc is a leptin concentration regulator in humans, and lack of it in the body leads to a decrease in the concentration of leptin [83]. In addition, zinc is a very important element for serotonin synthesis. Serotonin is a stimulator for the satiety sensation and reduction of appetite [83]. Zinc deficiency may harm the metabolism of hormones that have an important role in controlling and developing obesity [70].

Similarly, the mechanism for the effect of magnesium on obesity is not obvious, but according to the related studies, magnesium intake has an inverse relationship with hyperinsulinemia, metabolic syndrome, and reduction of the disposal of insulin-mediated glucose [84, 85]. The explanation for the mechanism by which selenium intake affects obesity is reported in the study of Azab et al. It is expressed that this mineral is a part of glutathione peroxidase, so it has substantial roles in controlling free radicals and further adverse reactive oxygen species that are related to obesity's metabolic complications [71].

The strength of our study is its design. This is an analytical cross-sectional study using baseline data of a well-stablished population-based cohort study with a large sample size; consequently, the risks of information and selection biases are lower with a cohort design. One limitation of this study could be incorrect in reporting of food intake due to self-reporting the FFQ.

## Figures and Tables

**Table 1:** Distribution of characteristics and dietary intakes across overweight, obese, and normal weight participants.

Characteristics	Overweight	Obese	Normal	*P* value^∗^	*P* value^∗∗^
Age	51.71 ± 8.3	51.3 ± 8.1	52 ± 8.27	<0.001	<0.001
Physical activity	37.88 ± 5.30	37.01 ± 4.11	39.67 ± 7.25	<0.001	<0.001
Education year	4.65 ± 4.58	3.94 ± 3.95	4.43 ± 4.55	0.02	<0.001
Gender				<0.001	<0.001
(i) Male	1689 (83.1)	343 (16.9)	2495 (59.5)		
(ii) Female	2667 (63.3)	15.49 (36.7)	1696 (40.5)		
Marriage status				0.09	<0.001
(i) Single	466 (61.3)	294(38.7)	402 (9.6)		
(ii) Married	3890 (70.9)	1598(29.1)	3789 (90.4)		
Has job				<0.001	<0.001
(i) Yes	2101 (78.2)	584(21.8)	2640 (63)		
(ii) No	2255 (63.3)	1308 (36.7)	1551 (37)		
Vitamin A (RAE)	0.79 ± 0.31	0.82 ± 0.32	0.75 ± 0.32	0.001	<0.001
Vitamin C (mg)	1.24 ± 0.52	1.31 ± 0.56	1.12 ± 0.51	<0.001	<0.001
Vitamin D	0.05 ± 0.03	0.05 ± 0.04	0.06 ± 0.03	<0.001	<0.001
Vitamin E (mg)	0.42 ± 0.13	0.43 ± 0.13	0.42±0.13	0.3	0.36
Vitamin B6 (mg)	5.52 ± 4.15	5.82 ± 5.30	5.36 ± 3.12	0.44	0.05
Vitamin B12 (mcg)	1.95 ± 1.09	1.90 ± 1.19	2.03 ± 1.20	0.03	0.06
Iron (mg)	1.26 ± 0.69	0.99 ± 0.53	1.55 ± 0.69	<0.001	<0.001
Folate	1.18 ± 0.22	1.15 ± 0.21	1.22 ± 0.22	<0.001	<0.001
Copper (mg)	±1.530.34	1.48 ± 0.33	1.56 ± 0.36	0.008	<0.001
Magnesium (mg)	±0.290.33	0.16 ± 0.26	0.45 ± 0.33	<0.001	<0.001
Zinc (mg)	0.9 ± 0.13	0.92 ± 0.14	0.86 ± 0.14	<0.001	<0.001
Calcium (mg)	0.86 ± 0.18	0.83 ± 0.17	0.88 ± 0.18	0.01	<0.001
Selenium (mcg)	1.58 ± 0.37	1.47 ± 0.33	1.67 ± 0.37	<0.001	<0.001
Thiamin (mg)	1.46 ± 0.29	1.43 ± 0.28	1.47 ± 0.30	0.32	<0.001
Riboflavin (mg)	1.33 ± 0.19	1.33 ± 0.19	1.32 ± 0.19	0.01	0.03
Niacin (mg)	1.34 ± 0.23	1.32 ± 0.23	1.35 ± 0.23	0.51	<0.001
Pantothenic acid	0.85 ± 0.20	0.83 ± 0.19	0.86 ± 0.22	0.11	<0.001
Total energy (kcal)	2381.57 ± 708.14	2306.30 ± 681.46	2449.67 ± 730.75	<0.001	<0.001
Physical activity	37.88 ± 5.30	37 ± 4.11	39.66 ± 7.25	<0.001	<0.001

^∗^ *P* value related to comparing characteristics and dietary intakes across overweight and normal weight participants. ^∗∗^*P* value related to comparing characteristics and dietary intakes across obese and normal weight participants.

**Table 2:** ORs and confidence intervals for the association between INQ and overweight (obesity) comparing normal weigh participants.

INQ	ORs for INQ (Overweight)	*P* value	ORs for INQ (obese)	*P* value
INQ vitamin A	0.98 )0.87, 1.08)	0.30	0.97(.86, 1.09)	0.33
INQ vitamin C	0.75 (0.54, 0.98)	0.02	0.73 (0.60, 0.89)	<0.001
INQ iron	0.60 (0.38, 0.95)	0.04	0.62 (0.38, 0.94)	0.03
INQ thiamin (b1)	0.74 (0.46, 0.89)	0.01	0.70 (0.49, 0.91)	0.11
INQ vitamin D	1 (0.88, 1.34)	0.68	1 (0.89, 1.30)	0.71
INQ vitamin E	0.58 (0.32, 0.80)	0.04	0.54 (0.35, 0.78)	0.01
INQ riboflavin (B2	0.92 (0.81, 1.03)	0.06	0.90 (0.77, 1.10)	0.07
INQ niacin (B3)	0.73 (0.60, 1.05)	0.17	0.74 (0.63, 1.21)	0.14
INQ folate (B9)	0.86 (0.59, 0.96)	<0.001	0.87 (0.55, 0.95)	<0.001
INQ B6	0.59 (0.41, 0.88)	0.03	0.51 (0.40, 0.85)	0.04
INQ vitamin B12	0.91 (0.79, 1.04)	0.65	0.90 (0.77, 1.06)	0.5
INQ pantothenic acid (B5)	0.77 (0.53, 1.03)	0.42	0.71 (0.60, 1.07)	0.43
INQ zinc	0.41 (0.31, 0.65)	0.01	0.40 (0.32, 0.64)	0.001
INQ selenium	0.87 (0.70, 1.01))	0.32	0.85(0.74, 1.09)	0.42
INQ magnesium	0.39 (0.28, 0.75)	0.04	0.32(0.23, 0.71)	0.02
INQ calcium	0.60 (0.51, 0.74)	<0.001	0.65(0.50, 0.76)	<0.001
INQ copper (cu)	1.02 (0.88, 2.04)	0.88	1.05(0.90, 2.07)	0.17

Adjusting for age and physical activity, gender, marital status, education year, and having job.

## Data Availability

The datasets used and analyzed during the current study are available by sending an email to the owner of data (Abbas Rezaianzadeh).
